# Transcatheter arterial chemoembolization combined with simultaneous DynaCT-guided microwave ablation in the treatment of small hepatocellular carcinoma

**DOI:** 10.1186/s40644-020-0294-5

**Published:** 2020-01-30

**Authors:** Zhaonan Li, Dechao Jiao, Xinwei Han, Guangyan Si, Yahua Li, Juanfang Liu, Yanneng Xu, Bo Zheng, Xun Zhang

**Affiliations:** 1 Department of Interventional Radiology, First Affiliated Hospital of Zhengzhou University, No. 1 Jianshe East Road, Zhengzhou City, 450000 Henan Province China; 2 Department of Interventional Radiology, The Affiliated Hospital of Traditional Chinese Medicine of Southwest Medical University, Luzhou, 646000 China

**Keywords:** Hepatocellular carcinoma, Microwave ablation, Interventional radiology, Transarterial chemoembolization

## Abstract

**Purpose:**

To evaluate the method and effectiveness of transcatheter arterial chemoembolization (TACE) combined with simultaneous DynaCT-guided Microwave ablation (MWA) for the treatment of small hepatocellular carcinoma (SHCC).

**Materials and methods:**

From June 2015 to May 2017, a total of 28 consecutive patients with SHCC received single treatment of TACE and 23 subjects received a combination treatment of TACE with simultaneous DynaCT-guided MWA. Following 1 month of treatment, the tumor response was assessed using the mRECIST criteria and the outcomes were analyzed including intervention-associated complications, changes in liver function, imaging response, and progression-free survival (PFS).

**Results:**

The technical success rate was 100%. The rates of CR (65%) in the combined TACE and MWA group were higher than those of the TACE group (46%). The rate of common adverse events, such as liver abscess, spontaneous bacterial peritonitis and liver dysfunction, in the combined TACE and MWA group (56%) was comparable to the corresponding rate of the TACE group (*P* > 0.411). The median and mean PFS of the TACE group were significantly lower than those of the combined TACE and MWA group (19.00 months vs. 29.00 months, 21.076 months vs. 24.693 months, *p* = 0.019, log-rank test).

**Conclusion:**

Stereotactic DynaCT-guided MWA is a safe and effective method for the treatment of SHCC, which usually provides an effective tumor puncture path, notably for lesions that cannot be detected following TACE. Overall, the data suggested that this treatment method could improve the clinical outcome of patients with SHCC.

## Introduction

Recent advances in image diagnostic technology and the widespread use of serological tests in high-risk populations have created potential opportunities for early clinical intervention in small hepatocellular carcinoma (SHCC) [1]. Although liver transplantation is still the ideal treatment for SHCC, only a small number of patients can receive this treatment [2–4]. In contrast to liver transplantation, surgical resection is considered to be the first-line treatment for SHCC [5–7]. Nevertheless, the risk of surgical resection for SHCC with multiple hepatic lobe lesions would undoubtedly increase. According to the Barcelona Clinical Liver Cancer (BCLC) guidelines, TACE is a palliative treatment modality. Of note, any single treatment has its own limitations and therefore, its efficacy may be limited [8]. In fact, Ablation (chemical or thermal) with TACE is established wherever the lipiodol deposition is incomplete and the lesion is targetable, and percutaneous acetic acid or ethanol injection is one of the important additional treatment options with TACE to improve outcome as it is cost effective [9–12]. Furthermore, the researchers revealed that MWA has recently gained importance and acceptance due to better tumor control as compared to radiofrequency ablation (RFA) [8, 13, 14]. The purpose of our retrospective study was to analyze the safety and efficacy of TACE combined with simultaneous DynaCT guided MWA by comparing the patient group that received single treatment with TACE for SHCC.

## Materials and methods

### Patients

The present retrospective study was approved by the institutional review board. The study protocol was based on the Helsinki Declaration. Written informed consent was obtained from all patients prior to treatment. Between June 2015 and May 2017, we retrospectively enrolled 23 patients in our institution who received TACE with simultaneous DynaCT-guided MWA for SHCC (median age 52 years; range 34–65 years) and 28 patients with SHCC who received conventional TACE alone (median age 56 years; range 36–69 years).

The inclusion criteria were the following: (a) Imaging or pathological examination for the confirmation of unresectable solitary HCC, or patient refusal to surgical resection; (b) patients aged 18 years or older; (c) HCC compliance with Milan’s HCC standard (single HCC nodules less than 5 cm in diameter or up to 3 nodules less than 3 cm in diameter) [1]; (d) Child–Pugh liver function grades of A and B, or BCLC grades of A and B; (e) an expected patient survival time higher than 3 months; (f) patients who received initial treatment; (f). The exclusion criteria were the following: (a) patients with severe coagulopathy; (b) presence of severe diseases (hepatic failure or heart failure); (c) severe portal hypertension (esophageal variceal bleeding, serious hypersplenism, or decompensated liver cirrhosis with ascites); (d) patients who did not receive multiple treatment options prior to intervention (DEB-TACE, conventional surgery, chemotherapy, or other anti-tumor treatment).

### Procedure

#### TACE treatment

Following routine skin sterilization, local anesthesia was performed with lidocaine. Subsequently, the modified Seldinger catheter was used for femoral artery puncture, and selective angiography of the superior mesenteric artery was performed to assess anatomical changes in the arteries and portal patency. Prior to TACE, iodine oil (Jiangsu Hengrui Medicine Co. Ltd., Jiangsu, China) was used as a carrier to load pirarubicin (THP; 60–80 mg; Shenzhen Meirui Pharmaceutical Co., Ltd. China). Hepatic artery angiography was performed using a 5-Fr catheter in order to identify the tumor and feeder(s). Superselective catheterization of the feeding artery was performed using a 2.0F microcatheter (Progreat, Terumo Corporation, Tokyo, Japan) followed by chemical embolization. Depending on the size and vascularity of the tumor, the amount of doxorubicin hydrochloride and iodized oil used was 10 to 20 mg (average 16.7 mg) and 4–7 ml (average 5.2 ml), respectively. Subsequently, 100–300 μm embolized microspheres (Jiangsu Hengrui Medicine Co. Ltd., Jiangsu, China) were used to embolize the blood supply until complete blood flow stasis. At the end of TACE, DynaCT (Artis zee BA Twin; Siemens AG, Germany; Syngo X-workplace with Syngo DynaCT; Siemens AG, Germany) was performed to evaluate the extent of Lipiodol uptake in the tumor.

#### MWA treatment

Following DynaCT completion and data acquisition and reconstruction, the iGuide mode (Syngo X-workplace with Syngo DynaCT; Siemens AG, Germany) was selected and the lesion was positioned in the axial, sagittal and coronal planes using a cross. DynaCT was used as the pre-designed procedure for lesion site puncture. After preparing the needle entry area in a sterile fashion, local anesthetic agent (lidocaine hydrochloride) was applied. Laparoscopic ultrasound (LUS; ALOKA ProSound α5, Aloka, Tokyo, Japan) was used during microwave ablation probe (ECO-100AI10, ECO Microwave System Co, Nanjing, China) insertion in order to avoid injury to the large vessels and the bile duct. When the optimal insertion angle and depth was achieved, the specific power and time settings were typically 5–10 min with a 65 watt (W) ablation [15]. The duration of ablation was directly related to the quality of the surrounding liver tissue, lesion depth and demarcation line length. Finally, the pre- and post-ablation CT scans were overlayed to validate the ablation zone.

### Evaluation of tumor response

Technical success was defined as the successful completion of TACE and MWA in one treatment session according to the modified response evaluation criteria in solid tumor (m-RECIST). The initial tumor response was assessed by contrast-enhanced MRI 1 month following treatment as follows: CR, complete response; PR, partial response; SD, stable disease; PD, progressive disease; objective response rate (ORR) = CR + PR; disease control rate (DCR) = CR + PR + SD.

### Follow-up

The laboratory tests were reviewed again within 1 week of treatment in order to assess the success of the procedure and to detect complications. One month after surgery, abdominal enhanced MRI/CT was performed to record the severity of lesion necrosis and local recurrence and the survival status was monitored during follow-up. In case of residual cancer, the secondary treatment with TACE-MWA or MWA alone would be performed separately according to the patient’s condition.

### Statistical analysis

The Chi-squared test or Fisher exact test were used to compare the categorical variables. Continuous variables were compared by the independent t-test. The categorical data were reported as numbers with percentages. PFS was estimated with the Kaplan–Meier survival analysis using the SPSS version 22.0 software (SPSS Inc., Chicago, IL). A *P* < 0.05 was considered for significant differences.

## Result

### Patient characteristics

**Fig. 1:**
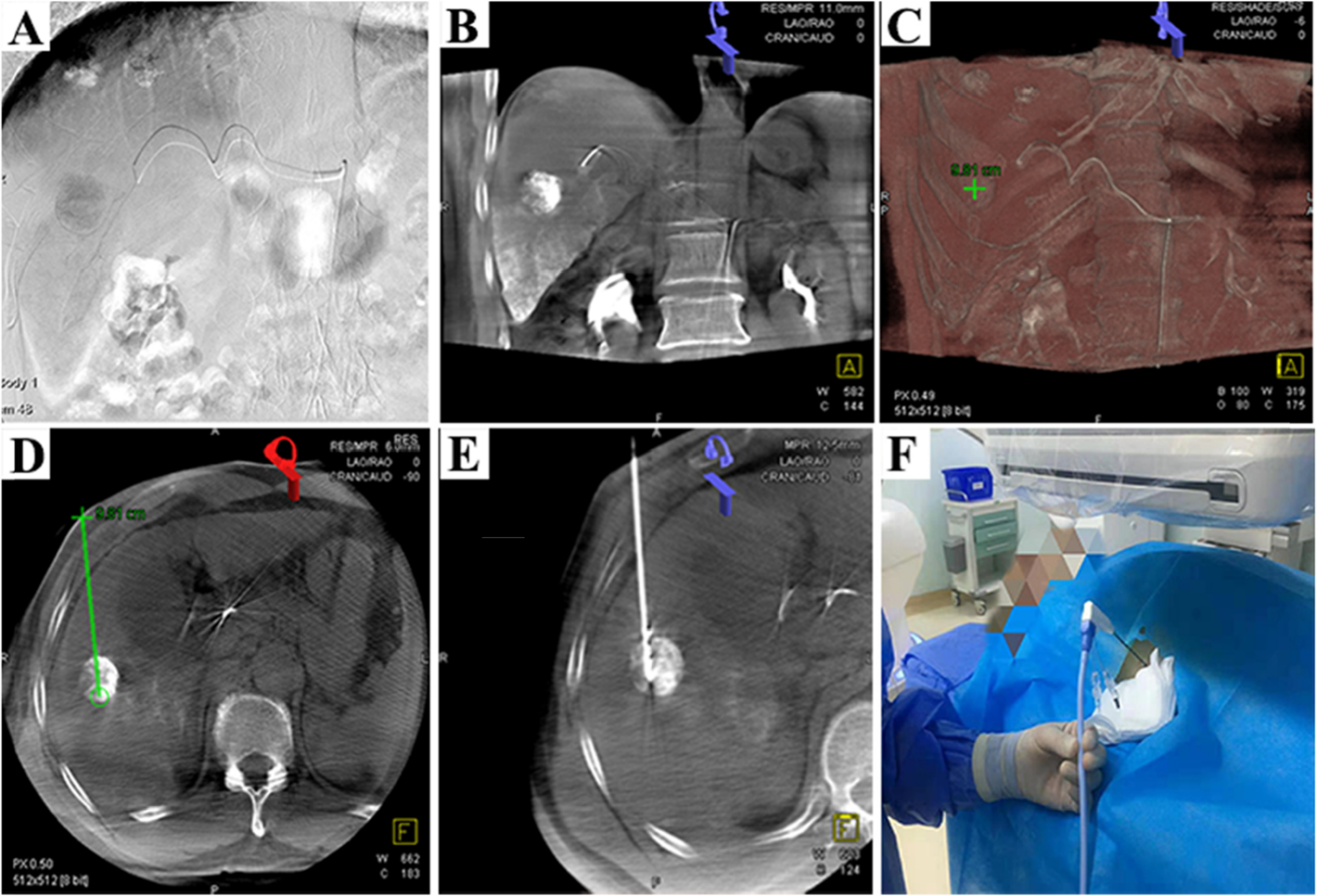
A 57-year-old male with SHCC, treated with TACE combined with simultaneous DynaCT-guided MWA. **a** Angiography demonstrated tumor-supplying arteries and embolized blood vessels with lipiodol. **b** Following TACE, a timely DynaCT scan revealed uneven deposition of lipiodol inside the tumor. **c** The application of the DynaCT workstation interface to resize the image and adjust the width and position of the window in order to clearly display the lesion. **d** The puncture was performed along the pre-designed routine under the guidance of DynaCT. **e**-**f** .The set power was 60 W for 12 min and the total duration of the three lesions was 30 min

**Fig. 2:**
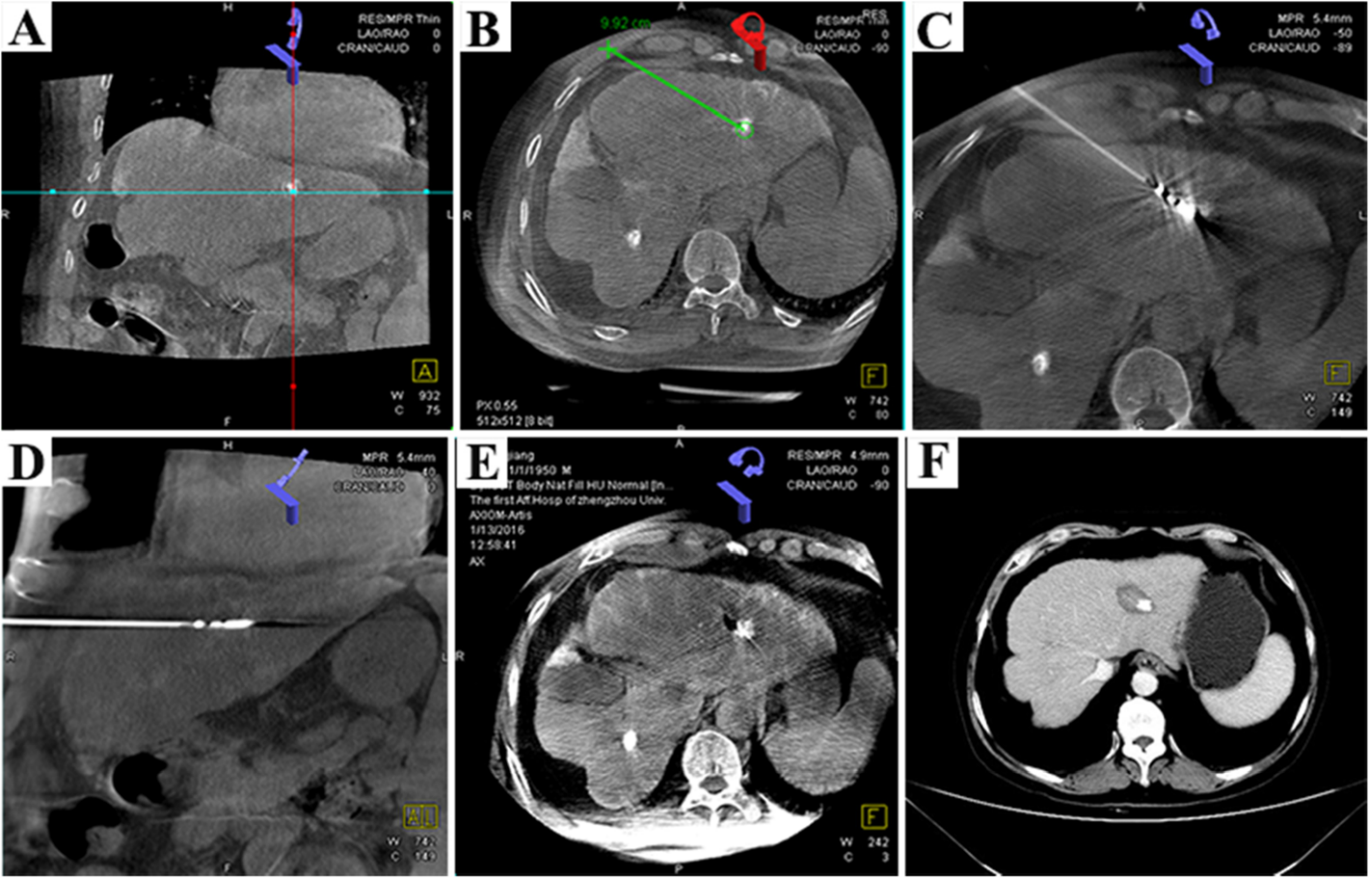
A 47-year-old male with SHCC, with a tumor of a minimum diameter of 1 cm, treated with TACE combined with simultaneous DynaCT-guided Microwave ablation (MWA). **a**-**e** The treatment resulted in partial tumor necrosis and additional bubble formation. **f**. Tumor necrosis in the TACE+MWA group after 1 month of treatment

This study retrospectively analyzed patients with SHCC recruited between June 2015 and May 2017. The baseline characteristics of the TACE and the TACE-MWA groups were well balanced **(**Table 1**)**. The technical success rate in both groups was 100%. The details of the interventional procedure in the combined treatment group are shown in **(**Fig. 1**)** and **(**Fig. 2**)**.

**Table 1:** Patient characteristics

Characteristics	TACE (*n* = 28)	TACE + MWA (*n* = 23)	*P* value
Age	52 (34–65)	56 (36–69)	0.231^b^
Sex			0.842^a^
Male	19 (68%)	15 (65%)	
Female	9 (32%)	8 (35%)	
ECOG performance status			1.00^c^
0	25 (89%)	21 (91%)	
1	3 (11%)	2 (9%)	
Etiology			0.487^c^
Hepatitis B	4 (14%)	1 (4%)	
Hepatitis C	16 (57%)	13 (57%)	
Alcohol	7 (25%)	6 (26%)	
Unknown	1 (4%)	3 (13%)	
Child–Pugh class			0.802^a^
A	18 (64%)	14 (61%)	
B	10 (36%)	9 (39%)	
Vascular invasion			
Portal vein (2nd or 3rd order)	2 (7%)	1 (4%)	
Hepatic vein	0	0	
AFP level	26.6 (2.9–655.2)	45.3 (4.2–876.7)	0.352
Tumor number			0.336^c^
Single	16 (57%)	8 (35%)	
2	9 (32%)	10 (43%)	
3	3 (11%)	4 (17%)	
> 3	0	1 (5%)	
Tumor diameter			0.254^a^
< 3	9 (32%)	11 (48%)	
3⩾,< 5	19 (68%)	12 (32%)	
Tumor location in liver			0.939^a^
Right lobe	6 (21%)	4 (17%)	
Left lobe	12 (43%)	10 (43%)	
Both lobes	10 (36%)	9 (39%)	

The data correspond to number of events

The data in parentheses correspond to percentages

^a^ Pearson chi-square test was used

^b^ Independent samples *t* test was used

^c^ Fisher exact test was used

### Local response

The treatment follow-up data are summarized in **(**Table 2**)**. Following 1 month of treatment, the tumor response was analyzed according to the definition of mRECIST. The rates of CR, PR, SD and PD in the TACE group were 13 (46%), 10 (36%), 5 (18%) and 0(0%), respectively. Therefore, the objective response rate (ORR) and DCR were 23 (82%) and 28 (100%), respectively, while, the rates of CR, PR, SD, PD in the TACE-MWA group were 15 (65%), 5 (22%), 4 (13%), 0(0%), respectively. The objective response rate (ORR) and DCR were 19 (83%) and 23 (100%), respectively (*P* = 0.437). The CR ratio of the TACE-MWA group was significantly higher than that of the conventional TACE group (65%vs. 46%).

**Table 2:** Follow-up data

Follow-up data	TACE (*n* = 28)	TACE + MWA (*n* = 23)	*P* value
mRECIST			0.437^a^
CR	13 (46%)	15 (65%)	
PR	10 (36%)	5 (22%)	
SD	5 (18%)	3 (13%)	
PD	0	0	
ORR	23 (82%)	19 (83%)	
DCR	28 (100%)	22 (100%)	
Child–Pugh class			0.764^b^
A	20 (71%)	15 (65%)	
B	8 (29%)	8 (35%)	
AFP level	5.2 (3.1–14.2)	4.9 (2.5–16.1)	0.011
Adverse Events			0.411^a^
Liver dysfunction	3 (11%)	1 (4%)	0.617^a^
Pleural effusion	1 (4%)	0	0.193^a^
Spontaneous bacterial peritonitis	2 (7%)	4 (17%)	0.390^a^
Gastrointestinal hemorrhage	0	0	n.d
liver abscess	5 (18%)	8 (35%)	0.207^a^
Pulmonary/cerebraloil embolization	0	0	n.d

*CR* complete response, *PR* partial response, *SD* stable disease, *PD* progressive disease; objective response rate (ORR) = CR + PR; disease control rate (DCR) = CR + PR + SD

The data correspond to number of events

The data in parentheses correspond to percentages

^a^Fisher exact test was used

^b^Pearson chi-square test was used. n.d, Not done

### Safety

**Fig. 3:**
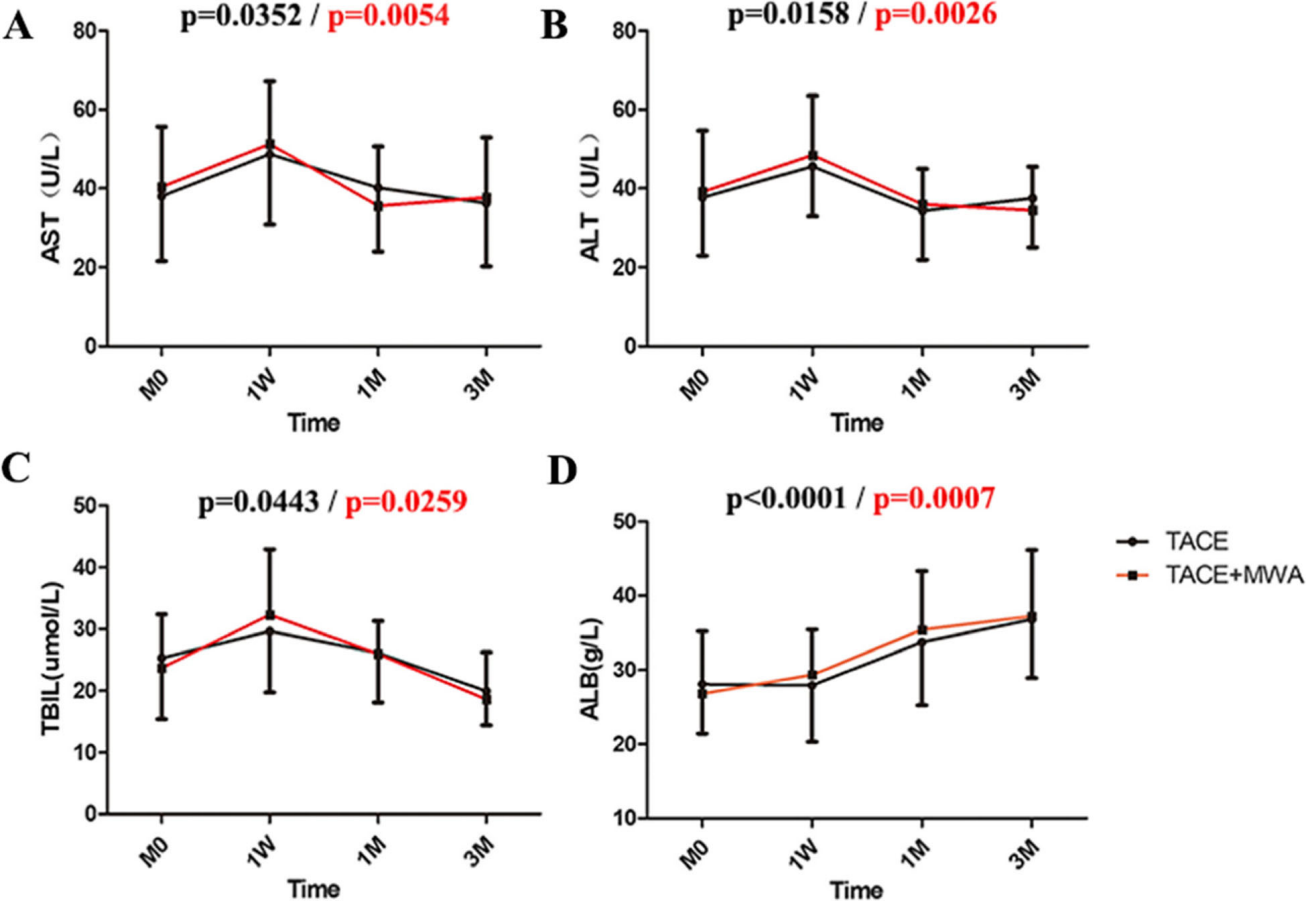
Changes in liver function at different times of treatment. **a**/**b** At 1 W, the levels of AST and ALT in the single TACE treatment group and in the TACE-MWA group increased significantly and gradually returned to the original level at 1 M and 3 M. **c** At 1 W, TBIL levels in the TACE group and the TACE-MWA group were significantly increased and continued to decrease following 1 M of treatment. **d** The ALB levels in the TACE group were decreased slightly within 1 week, while the ALB levels continued to increase following treatment in the TACE-MWA group. However, the two groups reached normal levels in M3. M0, pretreatment; 1 W, the first week following treatment; 1 M, the first month following treatment; 3 M, the third month following treatment. ALB, Albumin; AST, Aspartate aminotransferase; ALT, Alanine aminotransferase; TBIL, Total bilirubin

No significant differences were noted with regard to the adverse events between the two groups following 1 month of treatment (*P* > 0.05). The common adverse events in the TACE group were liver abscess, spontaneous bacterial peritonitis and liver dysfunction, with an incidence of 18, 11 and 7%, respectively. However, the common adverse events noted in the TACE-MWA group were liver abscess, gastrointestinal bleeding and liver dysfunction, with a probability of 35, 17 and 4%, respectively. Liver function tests **(**Fig. 3**)** revealed that aspartate aminotransferase (AST) and alanine aminotransferase (ALT) levels in both groups returned to preoperative levels at 3 months following treatment. The total bilirubin (TBIL) levels in the two groups indicated a downward trend and the albumin (ALB) concentration in both groups returned to normal levels following 3 months of surgery. The Child A and B grades of the TACE group were 71 and 29%, respectively, while those of the TACE - MWA group were 65 and 35%, respectively (*P* = 0.675).

### Progression-free survival (PFS)

**Fig. 4:**
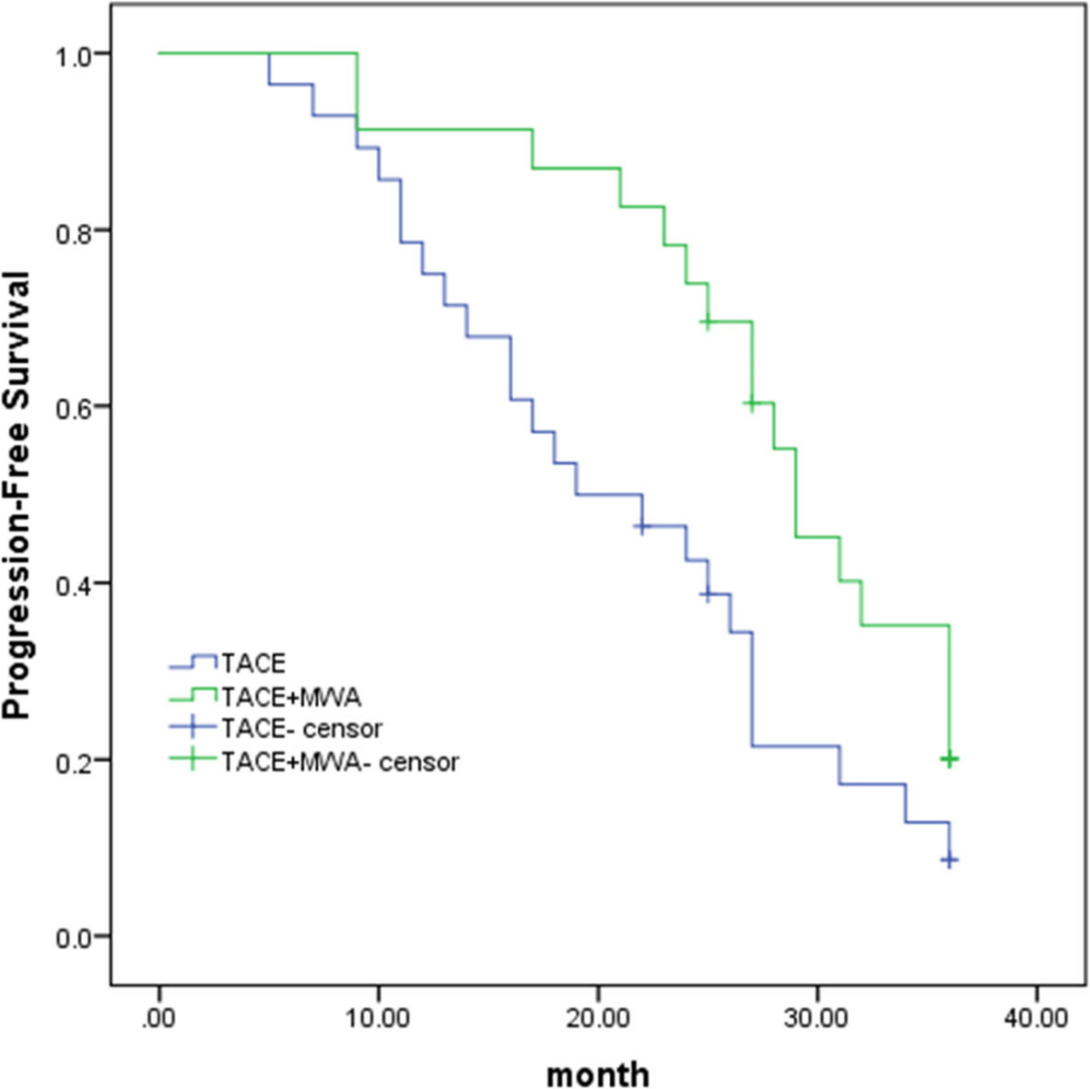
Kaplan–Meier progression-free survival (PFS) of the TACE alone group compared with the TACE-MWA group**.** The mean PFS was 21.076 months (95% CI:17.458, 24.693) in the TACE group compared with 28.216 months (95% CI: 24.793, 31.640) in the TACE-MWA group. The median PFS was 19.000 months (95% CI: 10.110, 27.890) in the TACE group compared with 29.000 months (95% CI: 24.786, 33.214) noted in the TACE-MWA group (*P* = 0.019, log-rank test)

The patients treated with TACE-MWA demonstrated higher PFS compared with that noted in the patients treated with conventional TACE monotherapy. The mean PFS was 21.076 months (95% CI: 17.458, 24.693) in the TACE group compared with 28.216 months (95% CI: 24.793, 31.640) noted in the TACE-MWA group, whereas the median PFS was 19 months (95% CI: 10.11, 27.89) in the TACE group compared with that noted in the TACE-MWA group (29 months, 95% CI: 24.786, 33.214) (*P* = 0.019, log-rank test) **(**Fig. 4**)**.

## Discussion

The results of the present study demonstrated that the combination of TACE with DynaCT-guided MWA could improve local tumor control in SHCC. The PFS of the TACE-MWA group was significantly higher compared with that noted in the conventional TACE group. In addition, the combination group did not increase the incidence of postoperative complications compared with that noted in the group that received single treatment with TACE.

MWA is a thermal ablation method. In comparison with RFA, MWA uses electromagnetic energy to achieve higher temperature in a shorter period of time and to produce larger ablation areas [16–19]. In a recent systematic review and meta-analysis, MWA was demonstrated to be as effective as RFA for HCC (OR 1.01, 95% CI 0.53–1.87, *p* = 0.98), and it provide superior outcomes compared with RFA in cases of larger nodules (OR 0.46, 95% CI 0.24–0.89, *p* = 0.02) [20] . Early studies have demonstrated the feasibility of TACE combined with MWA [21, 22]. A retrospective study of 244 HCC patients highlighted that the tumor response (CR + PR + SD) rates at 6 months was 92.1% in the TACE-MWA group compared with 46.3% noted in the conventional TACE (*P* < 0.001), and they concluded that patients with HCC tumors equal to or smaller than 5 cm in diameter (≤5 cm) exhibited a better response to combined treatment than that noted for TACE alone [23]. Additionally, the 1-, 3- and 5-year OS rates were significantly higher in the combination therapy group than those in the TACE alone group (*p* < 0.001) [24]. The mechanism of increased local tumor control that was noted in the combination group may be due to the complementarity of the two different therapies. Following TACE, the heat generated by the ablation needle could be concentrated in the lipiodol deposit, which could maximize the heat conduction effect of lipiodol and exert the maximum anti-tumor effect. In addition, the complex blood supply of certain irregular tumors led to uneven deposition of lipiodol. Therefore, MWA could target the ablation of residual tumor lesions and increase the rate of tumor necrosis. Furthermore, the use of MWA following lipiodol deposition can improve the transduction of heat to the peripheral tissues, thereby reducing recurrence and metastasis [25, 26].

Previous studies have suggested that the initial complete response represents an independent predictive factor for recurrence-free and disease-specific survival in patients undergoing ablation for HCC [24]. Moreover, the accurate placement of the ablation probes in the tumor targets is a crucial factor affecting HCC ablation [27]. Conventionally, sequential combination therapy is frequently adopted and ablation is usually performed 2–4 weeks following TACE under the guidance of computed tomography (CT) [14]. Recently, DynaCT employs cone-beam CT (CBCT) using the Artis zee DSA system (SIMENS, Germany) and allows for synchronized acquisition with flat-panel detector during C-arm rotation. And with the technique, TACE and MWA can be performed on the same working bed sequentially during one intervention. Following TACE, we now use DynaCT to scan the tumor and reconstruct 3D images for the puncture site and the route planning [28]. Wang et al. employed TACE combined with simultaneous CBCT -guided ablation to treat large HCCs in 21 patients, with a technical success rate of 100% [25]. In the study of Yuan et al., With precise guidance of CBCT, the ablation needle can accurately penetrate the target lesions of 46 patients and achieve favorable clinical results [29]. Therefore, DSA system with DynaCT allows for simultaneous combination of TACE and MWA in one intervention operation and more accurate puncture for ablation needles. And the synergism of these two benefits would further improve their therapeutic efficacy [30].

In the present study, the clinical effect of TACE combined with simultaneous DynaCT-guided MWA appeared superior to that caused by single application of TACE [31]. The progression-free survival of the combination treatment was significantly higher than that noted from single TACE treatment (*P* = 0.019). Following the combination treatment, the liver function of the patients was significantly altered and was gradually recovered to the preoperative level following 3 months of treatment, which was similar to the results reported by Andreano et al. [32]. Moreover, by adjusting the rack of the DynaCT machine, the time of patient transfer from the operating room to the CT room for MWA could be simplified, and the lipiodol deposition in the tumor tissue could be accurately displayed and recognized. This aided the decision making in the interventional procedures and resulted in improved safety of treatment by reducing the risk of operation.

Of note, the present study exhibits certain limitations. Initially, the sample size was relatively small that reduced the statistical power of the analysis. However, some of the results have already achieved the statistical significance. In addition, the short follow-up time period did not include the overall survival of the two groups and this may have led to biased results. It is also important to note that the process of puncture should be monitored continuously and may increase the radiation exposure of the clinician. Nevertheless, In order to minimize the side effects, we have tried to strictly monitor the radiation exposure time of clinician and patients, carefully evaluate the liver function reserve, and strive to improve the clinical prognosis of patients.

## Conclusion

Our preliminary study showed that TACE combined with DynaCT-guided MWA was safe and effective for the treatment of SHCC. The immediate combined therapy can achieve satisfactory short-term outcomes and benefit SHCC patients. However, additional prospective studies are required to further clarify its effectiveness.

## Data Availability

The datasets used and/or analyzed during the current study are available from the corresponding author on reasonable request.

## Abbreviations

ALB: Albumin
ALT: Alanine aminotransferase
AST: Aspartate aminotransferase
CBCT: cone-beam CT
CR: Complete response
DCR: Disease control rate
DEB-TACE: Drug-Eluting Bead Transarterial Chemoembolization
MWA: Microwave ablation
ORR: Objective response rate
PD: Progressive disease
PFS: Progression-free survival
PR: Partial response
RFA: Radiofrequency ablation
SD: Stable disease
SHCC: Small hepatocellular carcinoma
TACE: Transcatheter arterial chemoembolization
TBIL: Total bilirubin

## References

[1] Mazzaferro V, Regalia E, Doci R, Andreola S, Pulvirenti A, Bozzetti F (1996). Liver transplantation for the treatment of small hepatocellular carcinomas in patients with cirrhosis. N Engl J Med.

[2] Kamo N, Kaido T, Yagi S, Okajima H, Uemoto S (2016). Liver transplantation for small hepatocellular carcinoma. Hepatobiliary Surg Nutr.

[3] Zhu YB, Xu X, Zheng SS (2014). Association of microvascular invasion with recurrence and prognosis of patients with small hepatocellular carcinoma undergoing liver transplantation. Zhejiang Da Xue Xue Bao Yi Xue Ban.

[4] Mazzaferro V, Battiston C, Perrone S, Pulvirenti A, Regalia E, Romito R (2004). Radiofrequency ablation of small hepatocellular carcinoma in cirrhotic patients awaiting liver transplantation: a prospective study. Ann Surg.

[5] Feng Q, Chi Y, Liu Y, Zhang L, Liu Q (2015). Efficacy and safety of percutaneous radiofrequency ablation versus surgical resection for small hepatocellular carcinoma: a meta-analysis of 23 studies. J Cancer Res Clin Oncol.

[6] Huang G, Chen X, Lau WY, Shen F, Wang RY, Yuan SX (2014). Quality of life after surgical resection compared with radiofrequency ablation for small hepatocellular carcinomas. Br J Surg..

[7] Zhou J, Sun HC, Wang Z, Cong WM, Wang JH, Zeng MS (2018). Guidelines for Diagnosis and Treatment of Primary Liver Cancer in China (2017 Edition). Liver Cancer..

[8] Li W, Ni CF (2019). Current status of the combination therapy of transarterial chemoembolization and local ablation for hepatocellular carcinoma. Abdom Radiol (NY)..

[9] Huo TI, Huang YH, Wu JC, Chiang JH, Lee PC, Chang FY (2003). Sequential transarterial chemoembolization and percutaneous acetic acid injection therapy versus repeated percutaneous acetic acid injection for unresectable hepatocellular carcinoma: a prospective study. Ann Oncol..

[10] Huo T, Huang YH, Wu JC, Chiang JH, Lee PC, Chang FY (2004). Comparison of transarterial chemoembolization and percutaneous acetic acid injection as the primary loco-regional therapy for unresectable hepatocellular carcinoma: a prospective survey. Aliment Pharmacol Ther..

[11] Huang H, Liang P, Yu XL, Cheng ZG, Han ZY, Yu J (2015). Safety assessment and therapeutic efficacy of percutaneous microwave ablation therapy combined with percutaneous ethanol injection for hepatocellular carcinoma adjacent to the gallbladder. Int J Hyperthermia..

[12] Dong W, Zhang T, Wang ZG, Liu H (2014). Clinical outcome of small hepatocellular carcinoma after different treatments: a meta-analysis. World J Gastroenterol..

[13] Xu Z, Xie H, Zhou L, Chen X, Zheng S (2019). The Combination Strategy of Transarterial Chemoembolization and Radiofrequency Ablation or Microwave Ablation against Hepatocellular Carcinoma. Anal Cell Pathol (Amst).

[14] Liu Z, Gao F, Yang G, Singh S, Lu M, Zhang T (2014). Combination of radiofrequency ablation with transarterial chemoembolization for hepatocellular carcinoma: an up-to-date meta-analysis. Tumour Biol..

[15] Amabile C, Ahmed M, Solbiati L, Meloni MF, Solbiati M, Cassarino S (2017). Microwave ablation of primary and secondary liver tumours: ex vivo, in vivo, and clinical characterisation. Int J Hyperthermia..

[16] Goldberg SN, Gazelle GS, Mueller PR (2000). Thermal ablation therapy for focal malignancy: a unified approach to underlying principles, techniques, and diagnostic imaging guidance. AJR Am J Roentgenol..

[17] Liu FY, Yu XL, Liang P, Wang Y, Zhou P, Yu J (2010). Comparison of percutaneous 915 MHz microwave ablation and 2450 MHz microwave ablation in large hepatocellular carcinoma. Int J Hyperthermia..

[18] Liang P, Wang Y (2007). Microwave ablation of hepatocellular carcinoma. Oncology..

[19] Liang P, Dong B, Yu X, Yu D, Wang Y, Feng L (2005). Prognostic factors for survival in patients with hepatocellular carcinoma after percutaneous microwave ablation. RADIOLOGY..

[20] Facciorusso A, Di Maso M, Muscatiello N (2016). Microwave ablation versus radiofrequency ablation for the treatment of hepatocellular carcinoma: A systematic review and meta-analysis. Int J Hyperthermia..

[21] Yang WZ, Jiang N, Huang N, Huang JY, Zheng QB, Shen Q (2009). Combined therapy with transcatheter arterial chemoembolization and percutaneous microwave coagulation for small hepatocellular carcinoma. World J Gastroenterol..

[22] Seki T, Tamai T, Nakagawa T, Imamura M, Nishimura A, Yamashiki N (2000). Combination therapy with transcatheter arterial chemoembolization and percutaneous microwave coagulation therapy for hepatocellular carcinoma. Cancer-Am Cancer Soc..

[23] Chen QF, Jia ZY, Yang ZQ, Fan WL, Shi HB (2017). Transarterial Chemoembolization Monotherapy Versus Combined Transarterial Chemoembolization-Microwave Ablation Therapy for Hepatocellular Carcinoma Tumors &lt;/=5 cm: A Propensity Analysis at a Single Center. Cardiovasc Intervent Radiol..

[24] Xu LF, Sun HL, Chen YT, Ni JY, Chen D, Luo JH (2013). Large primary hepatocellular carcinoma: transarterial chemoembolization monotherapy versus combined transarterial chemoembolization-percutaneous microwave coagulation therapy. J Gastroenterol Hepatol..

[25] Wang ZJ, Wang MQ, Duan F, Song P, Liu FY, Chang ZF (2013). Transcatheter arterial chemoembolization followed by immediate radiofrequency ablation for large solitary hepatocellular carcinomas. World J Gastroenterol..

[26] Yuan H, Liu F, Li X, Guan Y, Wang M (2019). Angio-CT-Guided Transarterial Chemoembolization Immediately in Combination with Radiofrequency Ablation for Large Hepatocellular Carcinoma. Acad Radiol..

[27] Agopian VG, Harlander-Locke MP, Ruiz RM, Klintmalm GB, Senguttuvan S, Florman SS (2017). Impact of Pretransplant Bridging Locoregional Therapy for Patients With Hepatocellular Carcinoma Within Milan Criteria Undergoing Liver Transplantation: Analysis of 3601 Patients From the US Multicenter HCC Transplant Consortium. Ann Surg..

[28] Kato K, Abe H, Ika M, Yonezawa T, Sato Y, Hanawa N (2017). C-Arm Cone Beam Computed Tomography Guidance for Radiofrequency Ablation in Hepatocellular Carcinoma. Oncology..

[29] Yuan H, Liu F, Li X, Guan Y, Wang M (2019). Transcatheter arterial chemoembolization combined with simultaneous DynaCT-guided radiofrequency ablation in the treatment of solitary large hepatocellular carcinoma. Radiol Med..

[30] Wang ZJ, Wang MQ, Duan F, Song P, Liu FY, Wang Y (2013). Clinical application of transcatheter arterial chemoembolization combined with synchronous C-arm cone-beam CT guided radiofrequency ablation in treatment of large hepatocellular carcinoma. Asian Pac J Cancer Prev..

[31] Zhang TQ, Huang ZM, Shen JX, Chen GQ, Shen LJ, Ai F (2019). Safety and effectiveness of multi-antenna microwave ablation-oriented combined therapy for large hepatocellular carcinoma. Therap Adv Gastroenterol..

[32] Andreano A, Galimberti S, Franza E, Knavel EM, Sironi S, Lee FT (2014). Percutaneous microwave ablation of hepatic tumors: prospective evaluation of postablation syndrome and postprocedural pain. J Vasc Interv Radiol..

